# Optimized Chitosan-Based Nanoemulsion Improves Luteolin Release

**DOI:** 10.3390/pharmaceutics15061592

**Published:** 2023-05-25

**Authors:** Camila Diedrich, Isabella C. Zittlau, Najeh M. Khalil, Adam F. G. Leontowich, Rilton A. de Freitas, Ildiko Badea, Rubiana M. Mainardes

**Affiliations:** 1 Pharmaceutical Nanotechnology Laboratory, Universidade Estadual do Centro-Oeste, Guarapuava 85040-167, Brazil; camiladiedrich@hotmail.com (C.D.); isazittlau99@gmail.com (I.C.Z.); najeh@unicentro.br (N.M.K.); mainardes@unicentro.br (R.M.M.); 2 Canadian Light Source, Saskatoon, SK S7N 2V3, Canada; adam.leontowich@lightsource.ca; 3 Biopol, Chemistry Department, Federal University of Parana, Curitiba 81531-980, Brazil; 4 Drug Design and Discovery Research Group, College of Pharmacy and Nutrition, University of Saskatchewan, Saskatoon, SK S7N 5E3, Canada

**Keywords:** optimization, chitosan, stability, response surface methodology, drug release

## Abstract

Luteolin (LUT) is a flavonoid found in several edible and medicinal plants. It is recognized for its biological activities such as antioxidant, anti-inflammatory, neuroprotective, and antitumor effects. However, the limited water solubility of LUT leads to poor absorption after oral administration. Nanoencapsulation may improve the solubility of LUT. Nanoemulsions (NE) were selected for the encapsulation of LUT due to their biodegradability, stability, and ability to control drug release. In this work, chitosan (Ch)-based NE was developed to encapsulate luteolin (NECh-LUT). A 2^3^ factorial design was built to obtain a formulation with optimized amounts of oil, water, and surfactants. NECh-LUT showed a mean diameter of 67.5 nm, polydispersity index 0.174, zeta potential of +12.8 mV, and encapsulation efficiency of 85.49%. Transmission electron microscopy revealed spherical shape and rheological analysis verified the Newtonian behavior of NECh-LUT. SAXS technique confirmed the bimodal characteristic of NECh-LUT, while stability analysis confirmed NECh-LUT stability when stored at room temperature for up to 30 days. Finally, in vitro release studies showed LUT controlled release up to 72 h, indicating the promising potential of NECh-LUT to be used as novel therapeutic option to treat several disorders.

## 1. Introduction

**Figure 1:**
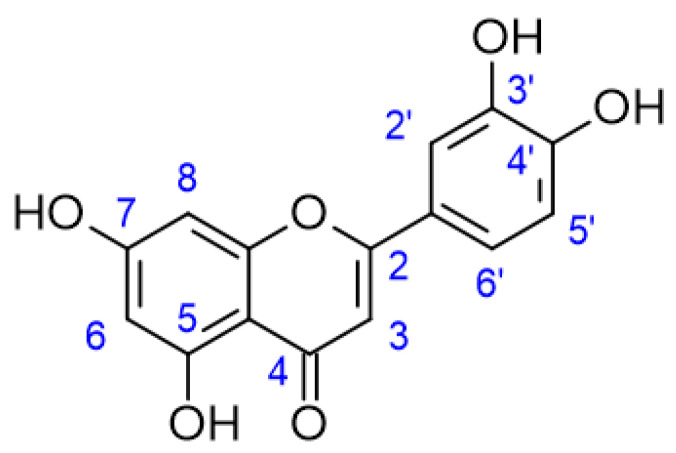
Luteolin structure.

Luteolin (5,7,3′,4′-tetrahydroxyflavone) (LUT) is a flavonoid commonly found in plants such as carrot, pepper, mint, thyme, rosemary, oregano, lettuce, pomegranate, cucumber, among others [1]. LUT presents a C6-C3-C6 flavonoid structure added by two benzene rings (B, C), a third oxygen-containing (C) ring, a double bond between carbons 2 and 3, a carbonyl group on carbon 4, and hydroxyl groups at carbons 5, 7, 3′ and 4′, as shown in Figure 1 [2,3].

The hydroxyl moieties and 2–3 double bond in LUT structure plays an important role on its pharmacological properties [3,4]. The 3′, 4′ hydroxylation, the presence of a double bond between carbons 2 and 3, and a carbonyl group on carbon 4 are essential to LUT activities since hydrogens linked to the aromatic hydroxyl group are easily donated to stabilize free radicals [3]. Among the biological activities of LUT, the most prominent are the neuroprotective, antinociceptive, antioxidant, antimicrobial, antiviral, and antitumor effects [5,6,7,8,9,10,11]. Although the pharmacological activities of LUT had been confirmed by several studies, its low solubility in water leads to pharmaceutical limitations due to low absorption by the body and low bioavailability [12]. Studies demonstrate that after oral administration, LUT is rapidly absorbed and metabolized, giving rise to eight metabolites with short half-lifes, which are distributed in the body and excreted in the bile, the main route of LUT excretion [13].

Nanoemulsions (NE) are emulsions smaller than 200 nm, transparent in appearance and kinetically stable, but not thermally stable [14]. The kinetic stability of NE is due to the fact that the Brownian motion overcomes the gravitational forces between the phases of the NE, which prevents the droplets from aggregating [15]. Among the advantages of using NE for drug delivery are improved absorption, permeability, and stability, as well as biocompatibility, biodegradability, and controlled release. These properties, allowing dose reduction, lead to reduced side-effects [14]. According to the proportion of water and oil and nature surfactants, oil-in-water (O/W) or water-in-oil (W/O) NE form [16,17]. An O/W type NE is obtained by dispersing the oil phase in the aqueous phase, stabilized by the surfactant [18]. Considering that most bioactive compounds with pharmacological potential are hydrophobic, O/W-type NE is commonly used as a means of increasing their water solubility [19]. The stability of NE is a determining factor for their commercial application. Processes such as flocculation, coalescence, and Ostwald ripening are the most common destabilization processes in NE [17]. Flocculation occurs through the junction of NE globules that form larger droplets, which rise or settle, forming a creamy-looking layer [20]. Coalescence occurs by the rupture of the continuous surfactant film on the NE globules, leading to fusion with other larger globules [18]. Finally, Ostwald ripening is a destabilization process that occurs through the diffusion of smaller droplets in the aqueous medium of larger droplets, promoting size increase [21].

Chitosan (Ch) is an amino polysaccharide composed of N-acetyl and glucosamine units linked in β-(1-4) configuration, produced through the deacetylation of chitin from the insect cuticle and exoskeleton of crustaceans and cephalopods [22]. Due to its positive charge, mucoadhesion, film-forming, and low-cost characteristics, Ch is one of the most applied biopolymers in the food and pharmaceutical industry [23]. In addition, Ch has been widely used in medicine due to its biocompatibility, biodegradability, and low toxicity, being approved by the USA Food and Drug Administration (FDA) for the production of several formulations [24]. When added in NE, Ch improves the mechanical properties of NE through electrostatic interactions and increasing the stability of the emulsified system [25]. Therefore, this study aimed to produce and characterize an optimized chitosan-based NE containing LUT and verify its improved drug release.

## 2. Materials and Methods

### 2.1. Materials

Luteolin (>98% purity) was purchased from Vicofarma^®^ (São Paulo, Brazil). Quercetin (IS) (>98% purity) and 75–85% deacetylation degree low molecular weight chitosan (50,000–190,000 Da) were purchased from Sigma Aldrich^®^ (St. Louis, MO, USA). HPLC-grade formic acid and methanol were purchased from Merck^®^ (Darmstadt, Germany). HPLC-grade acetonitrile (ACN) was purchased from LiChrosolv^®^ (Darmstadt, Germany). Monobasic potassium phosphate, sodium pyrophosphate, and oleic acid were purchased from Synth^®^ (Diadema, Brazil). Hydrochloric acid and acetic acid were purchased from Vetec^®^ (Duque de Caxias, Brazil). Ketamine and xylazine were purchased from Ceva^®^ (Paulinia, Brazil). Sodium chloride, sodium hydroxide, ethylene glycol, and dibasic sodium phosphate were purchased from Biotec^®^ (Lages, Brazil). Potassium chloride was purchased from Química Moderna^®^ (São Paulo, Brazil). Tween 20 was purchased from Neon Comercial^®^ (Suzano, Brazil). Ultrapure water was obtained by Millipore^®^ purification system (Burlington, MA, USA).

### 2.2. LUT Solubility Studies

The solubility of LUT in NE oil and surfactants was determined by UV-ViS spectroscopy. A total of 10 mg of LUT was weighed and added to 1 mL of each component (oleic acid, Tween 20 and ethylene glycol). The mixture was stirred at 300 rpm for 24 h, at 37 °C, and centrifuged at 12,000 rpm for 5 min. The supernatant was collected, and reading was performed on UV-Vis equipment at 263 nm. Analyses were performed in triplicate using an analytical curve of LUT at concentrations between 5 and 60 µg/mL.

### 2.3. Preparation of Nanoemulsion

NE was produced employing cavitation method. The aqueous phase was dropped into the oil phase during sonication. The mixture was stirred in a sonicator (Unique^®^, Indaiatuba, Brazil) for three cycles of 1 min at 30 s intervals, applying 90% power. The oil phase was composed of oleic acid as oil and ethylene glycol and Tween 20 as surfactants, while the aqueous phase was composed of chitosan solubilized in 0.25% acetic acid. The surfactants ratio was determined by the modulation of the hydrophilic–lipophilic balance (HLB) in order to obtain the most suitable proportion of surfactants, considering LUT solubility and HLB value between 8 and 18, consistent with the formation of oil in water NE.

### 2.4. Nanoemulsion Optimization

A 2^3^ factorial design was carried out to evaluate the effects of the amounts of oil, surfactant mixture, and aqueous phase on the mean diameter and polydispersity index of the NE. The design consisted of eight runs in triplicate, the volumes of oil (X1), aqueous phase (X2) and surfactants (X3) being the independent variables, tested at two levels, −1 and +1. The dependent variables were mean diameter (Y1) and polydispersity (Y2). Table 1 describes the 2^3^ factorial design performed for the NE optimization.

### 2.5. Preparation of Chitosan-Based Nanoemulsion

After the determination of the optimized conditions for preparing the NE, Ch was added in the aqueous phase at different concentrations (0.10; 0.25; 0.5, and 1.0%).

### 2.6. Nanoemulsion Characterization

#### 2.6.1. Mean Diameter and Zeta Potential Analysis

The mean diameter (D) and polydispersity index (PDI) of the NE were measured by the photon correlation spectroscopy or dynamic light scattering (DLS) technique (Brookhaven 90 Plus^®^, New York, NY, USA). The samples were diluted in ultrapure water (1:100) and placed in a cuvette with a lid. The analyses were performed with a scattering angle of 90°, at 25 °C, and laser wavelength of 659 nm. The measurements were performed in triplicate and results expressed as mean ± standard deviation.

The analysis of the Zeta potential (ZP) was performed by determining the electrophoretic mobility of the NE using the ZetaSizer device (ZS-Malvern^®^, Cambridge, UK). The samples were diluted (1:100) in 1 mM KCL aqueous solution and placed in the electrophoretic cell, where a potential of ±150 mV was established. The determined ZP values were expressed as mean ± standard deviation and the analyses were performed in triplicate.

#### 2.6.2. Encapsulation Efficiency

The encapsulation efficiency (EE) of the NECh-LUT was determined directly by extracting LUT from NE formulation using methanol (25:200). The samples were centrifuged at 25,000 rpm, 24 °C, for 45 min, and an aliquot of the supernatant was filtered into a 0.45 µm (polyvinylidene fluoride) PVDF hydrophobic filter (Millipore^®^, Burlington, MA, USA) and quantified by high-performance liquid chromatography coupled to a photodiode array detector (HPLC-PDA).

The chromatographic separation for LUT quantification was performed using a HPLC chromatograph (Model e2695, Waters^®^, Milford, MA, USA) coupled to a photodiode array detector (Model 2998, Waters^®^, Milford, MA, USA) and equipped with Empower Pro software (Waters^®^, Milford, MA, USA). A C18 reverse phase column (250 × 4.6 mm) with 5 µm pore (LiChrospher^®^ RP18-5) was employed in the separation. The isocratic mobile phase consisted of acetonitrile and 0.5% (*v*/*v*) formic acid (45:55) in the flow of 1 mL/min for the total running time of 5 min. A 20 µL sample volume was injected in the column maintained at 30 °C. LUT was identified at a wavelength of 357 nm and retention time of 4.28 min [26,27,28,29].

#### 2.6.3. Morphology

The morphological analysis of NECh-LUT was performed using transmission electron microscopy (TEM) in a model HT7700 Hitachi^®^ (Tokyo, Japan). The samples were diluted in ultrapure water (1:100) and placed on a 300-mesh copper grid coated with formvar and fixed with 1% phosphotungstic acid for 30 s. The excess was removed using filter paper and the grids were dried at room temperature for 2 h. The micrographs were obtained using a voltage of 100 kV.

#### 2.6.4. Rheology

The dynamic oscillatory analysis was performed using Thermo Scientific Haake Rheostress 1 system (Karlsruhe^®^, Karlsruhe, Germany) and RheoStress software, equipped with cone and plate geometries with a 60 mm diameter. The viscosity analyses were performed in an ascending ramp at a shear rate from 4 to 50 s^−1^ with duration of 300 s and descending ramp at shear rate from 50 to 0 s^−1^, both at 22 °C.

#### 2.6.5. Small Angle X-ray Scattering

Small angle X-ray scattering (SAXS) measurements were performed using the wiggler low energy (WLE) beamline [30] at the Canadian light source (CLS) synchrotron facility (Saskatoon, Canada). The endstation was set to operate at a photon energy of 10,491.4 eV, energy resolution of 3.88 eV, beam size FWHM of 125 µm × 350 µm, and flux of 4.1 × 10^11^ photons/s on the sample. A Rayonix MX300 detector (300 mm × 300 mm active area with 73.242 μm × 73.242 μm pixels), 2.3 m from the sample, was used to record the SAXS patterns as 2D images. A 2.2 m long flight tube with a 200 nm thick silicon nitride entrance window, a 4.0 mm diameter active beamstop, and 125 µm thick kapton exit window was positioned between the sample and detector. Samples were loaded into 1.5 mm internal diameter borosilicate capillaries (Charles Supper Company©, Westborough, MA, USA) and fixed in the sampler, and then exposed to X-rays for 300 s. The 2D detector images were calibrated against a silver behenate standard, blank subtracted, and reduced to 1D intensity (I) versus momentum transfer (q, A^−1^) plots all using the program GSAS-II, version 5329 [31]. The measurements cover a q range of 0.008–0.35 A^−1^ [31,32]. SAXS data was fit using the modeling tool within the Irena package, version 2.71 [33].

### 2.7. Stability Studies

NECh and NECh-LUT formulations were evaluated for kinetic, thermal, and storage stability. The kinetic stability of the samples was performed by centrifugation in a refrigerated ultracentrifuge (Cientec^®^, Belo Horizonte, Brazil) at 5000 rpm and 25 °C for 30 min [34]. Thermal stability was evaluated by heating the samples at 50 °C for 30 min [35] and freezing at −20 °C for 24 h. Storage stability was tested at room and refrigeration temperature for 30, 60, and 90 days [36]. The NE stability was determined in triplicate through macroscopic observation, followed by the measurement of the Dm and ZP.

### 2.8. In Vitro Release

LUT release from NECh-LUT was evaluated using a Franz diffusion cell (Teledyne Hanson Research^®^, Chatsworth, CA, USA). Prior to the analysis, a 0.45 µm nitrocellulose membrane (Merck Millipore^®^, Dublin, Ireland) was kept in PBS pH 7.4 for 20 min to equilibrate, and then fixed to the diffusion cell. The receptor chamber was filled with 7 mL of PBS and maintained under magnetic stirring at 300 rpm and 37 °C. An aliquot of 0.175 mL of NECh-LUT was added to the donor chamber and the compartments were closed. At pre-determined times (0.25, 0.5, 0.75, 1, 2, 4, 8, 12, 24, 48, 60, and 72 h), samples of 1 mL were withdrawn from the receptor chamber and then replaced by fresh PBS. The samples were filtered through 0.45 µm PVDF hydrophilic filters and analyzed by HPLC-PDA as described in Section 2.6.2. The analysis was carried out in triplicate and a cumulative amount of drug released versus time was plotted to obtain the release kinetics model. The kinetic mechanism of LUT release was studied using the correlation coefficient (r^2^) of both formulation and employing the mathematical models of zero, first and second order, Higuchi, Hixson–Crowell, and Weibull using free software KinetDS 3.0 (Faculty of Medicine, Jagiellonian University, Krakow, Poland).

### 2.9. Statistical Analysis

Statistical analysis of the data obtained from the 2^3^ factorial design used was performed using one-way analysis of variance (ANOVA). Experimental data were analyzed to fit second-order polynomial regression containing linear and quadratic coefficients and two-factor interaction effects. Models and regression coefficients were considered significant when *p* < 0.05. Pareto charts and the response surface methodology (RSM) were used to assess the effects of factors on the dependent variables. Experimental data were expressed as mean ± standard deviation of at least three replicates. One-way ANOVA followed by Tukey test was used to compare means at the 95% confidence level of statistical significance, *p* < 0.05. The Statistica software (StatSoft Inc^®^, Tulsa, OK, USA) was employed for the statistical analyses.

## 3. Results and Discussion

### 3.1. LUT Solubility Studies and Hydrophile–Lipophile Balance Manipulation

In the process of obtaining a NE, one must consider the toxicity of the components, the solubility of the drug in the vehicle, and the physicochemical characteristics of these components [35]. LUT solubility was found to be 2.82, 7.09, and 6.26 mg/mL in oleic acid, ethylene glycol, and Tween 20, respectively. LUT solubility in ethylene glycol is in agreement with that found in the literature [37]. For Tween 20 and oleic acid, data on the solubility of LUT in these components were not found. Considering LUT solubility, ethylene glycol and Tween 20 were chosen as solubilizing agents in the NE formulation. The manipulation of HLB was performed in order to provide a value consistent with O/W NE. This way, the proportion of 25:75 ethylene glycol: Tween 20 was employed, resulting in an HLB value of 13.

### 3.2. Nanoemulsion Optimization

LUT-containing NE was optimized through the evaluation of the independent variables (oil, aqueous phase, and surfactants volumes) on the dependent variables (mean diameter and polydispersity index). As shown in Table 2, the Dm ranged from 39.7 for formulation 4 to 2389.7 nm for formulation 5, and the PDI ranged from 0.263 to 0.975.

Table 3 presents the ANOVA values for the dependent variables Dm and PDI. Dm was significantly influenced by oil, aqueous phase, and surfactant factors, and by the interaction between the factors (*p* < 0.05). Additionally, the PDI was influenced by the aqueous phase, surfactant, and the interactions between these factors (*p* < 0.05). The oil volume did not influence significantly PDI; however, the interaction between oil volume and the other factors had statistically significant influence on the PDI (*p* < 0.05).

**Figure 2:**
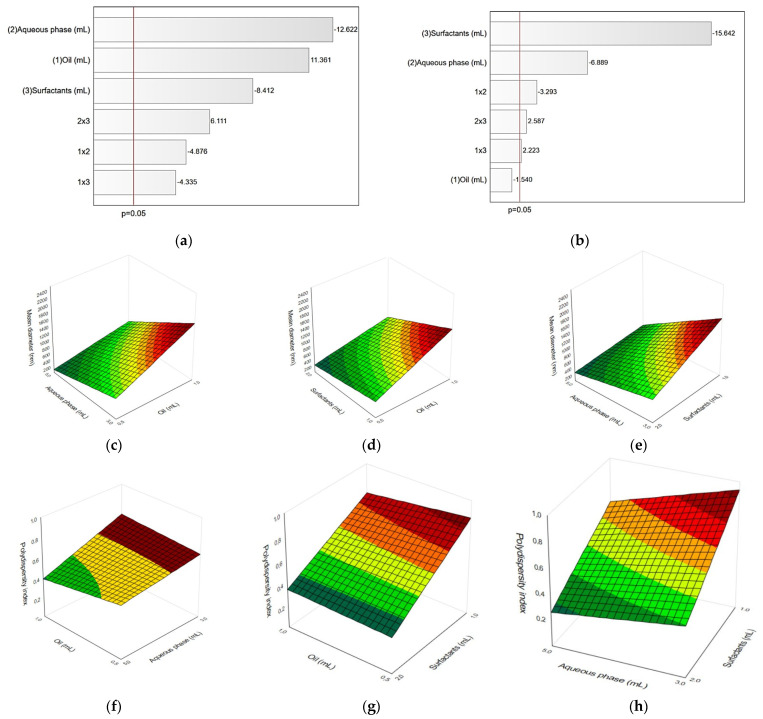
Pareto charts (**a**,**b**) and response surface graphs (**c**–**h**) for the dependent variables mean diameter and polydispersity index.

All isolated factors and interactions between factors were significant for Dm, except the oil volume did not significantly influence PDI (Table 3). Pareto charts show the effects of oil, aqueous phase, and surfactants factors and the interaction between them on Dm and PDI, from the largest to the smallest effect, passing through the statistical significance line (*p* < 0.05), as shown in Figure 2a,b. The volume of oil was the only non-significant factor for PDI. For Dm, aqueous phase volume was the factor that most interfered in the response (−12.622). The negative value indicates that the greater the volume of the aqueous phase, the lower the Dm. The oil volume positively interfered in the Dm (11.361). The volume of the surfactant mixture had a negative influence on Dm (−8.412), indicating that the larger the number of surfactants, the smaller the Dm. In addition, the interaction between aqueous phase and surfactant volumes had the greatest effect in Dm (6.111), followed by the interactions between oil and aqueous phase (−4.876) and between oil and surfactants volume (−4.335). The greatest negative influence exerted on PDI was by the surfactant volume (−15.642) and aqueous phase volume (−6.889), indicating that an increase in the amount of aqueous phase and/or surfactants relates to lower PDI values. Additionally, the interaction between oil and aqueous phase (−3.293), aqueous phase and surfactant (2.587), and oil and surfactants (2.223) were significant regarding the PDI effect, while oil volume did not influence this result.

The results obtained for NE formulations were also analysed through multiple linear regression, generating adjusted models, taking into account only the significant effects for the dependent variables Dm and PDI, as shown in Table 4. The values of the correlation coefficients above 0.75 indicate that the applied models fit the experimental data.

From the linear regression models, response surface graphs were generated, as shown in Figure 2c–h. On the response surfaces, the dependent variables Dm and PDI are presented on the Z axis as a function of the independent variables oil phase, aqueous phase, and surfactants volume. For Dm, smaller volumes of oil and higher volumes of aqueous phase and surfactants proved to be the most beneficial to obtain smaller NE droplets. For PDI, higher volumes of aqueous phase and surfactants produced uniform NE, with the oil volume non-relevant regarding this response. The overall observations indicate that aqueous phase content was the most important factor influencing Dm, while surfactants content was the most influential factor affecting PDI. According to Alzorqi et al., an increase in water content leads to the decrease in Dm of NE due to higher surfactant adsorption and surface activity in the oil–water interface [36]. Sood et al. postulates that higher surfactant amounts result in sufficient coverage of oil droplets, reducing interfacial between oil and water [38].

From the analysis of the ANOVA, Pareto charts and response surface analyses, the NE formulation was established with levels −1 for the volume of oil and +1 for the volumes of aqueous phase and mixture of surfactants, which together describe Formulation 4. Formulation 4 is composed of 0.5 mL of oil, 5.0 mL of aqueous phase, and 2.0 mL surfactants (Table 2). This composition with 0.8 mg/mL LUT was employed for the continuation of the study.

### 3.3. Chitosan Coating

The contribution of chitosan (Ch)-adding to Dm, PDI, and ZP was assessed (Table 5). Dm and ZP increased proportionally with higher concentrations of Ch. When the concentration of Ch increased from 0 to 1.0% (*w*/*v*), the Dm increased from 39.7 to 378.5 nm, while PDI increased from 0.136 to 0.334. The ZP presented values from −3.7 to +25.3 mV when the concentration of Ch varied from 0 to 1.0% (*w*/*v*). The addition of Ch provided a ZP change from negative to positive values. The increase in Dm, PDI, and ZP values was also reported by other authors. Shah et al. observed the increase in Dm, PDI, and ZP of the Ch-coated NE and attributed the increase in ZP to the cationic characteristic of Ch [39]. Silva et al. evaluated the change in Dm, PDI, and ZP parameters from the addition of different concentrations of Ch, verifying the increase in both parameters after the addition of the polymer [40]. Considering the results of Dm, PDI, and ZP, the concentration of 0.25% of Ch (Formulation 10) was considered the most appropriate to continue the experiments due to Dm smaller than 100 nm, droplets uniformity, and positive ZP.

### 3.4. Nanoemulsion Characterization

#### 3.4.1. Globule Size and Zeta Potential Analysis

**Figure 3:**
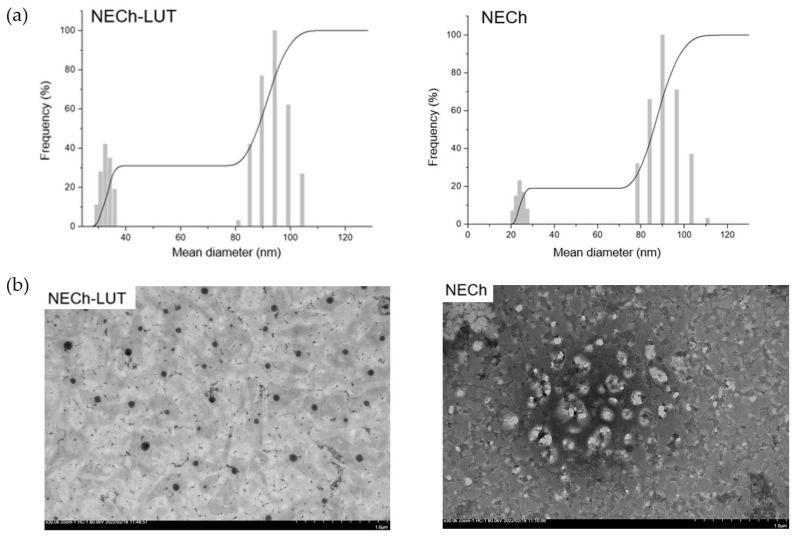
Mean diameter distribution determined by (**a**) DLS and (**b**) TEM micrography of NECh-LUT and NECh formulations at 30.000 × zoom.

The empty NE with Ch (NECh) presented a clear and viscous aspect, while the LUT-loaded NE without Ch (NE-LUT) presented a translucent aspect with a slight yellowish color. The LUT-loaded, Ch-containing NE (NECh-LUT) also presented the yellow color from LUT. Table 6 shows Dm, PDI, and ZP results of NECh, NECh-LUT, and NE-LUT formulations. NECh-LUT and NECh did not present statistical difference in the Dm of 67.5 and 61.8, respectively, while NE-LUT presented smaller size (37.7 nm) when compared to the formulations without Ch (*p* < 0.05). The larger size of Ch-containing NE was expected due to the presence of an outer layer composed of Ch [41]. NECh-LUT showed a bimodal pattern, characteristic of NE prepared using the cavitation method (Figure 3a) [18]. The ZP indicates the NE surface charge and directly impacts the mucoadhesion due to the interaction with negatively charged mucin present in mucous [42]. Additionally, ZP values distant from zero prevent the NE coalescence process [43]. NE-LUT presented ZP of −3.7 mV, while NECh-LUT and NECh presented values of +12.8 and +10.6, respectively. The positive value of ZP after Ch-adding demonstrates that the addition of Ch to the aqueous phase was enough to invert ZP in the formulations. NE smaller than 100 nm have a transparent appearance, as their droplets are smaller than the wavelength of visible light [44]. Alzorqi et al. attributed the small diameter of the NE produced by ultrasound to the intensity of the cavitation process and the ability of the surfactant to decrease the surface tension of the droplets [36]. In fact, the cavitation process is recognized for its ability to reduce interfacial tension and prevent droplet coalescence [45], in addition to reducing NE Dm [45,46]. In addition, the use of co-emulsifier such as ethylene glycol in the NE formulation provides the interfacial film fortification, as they fit between structurally weaker areas [47]. Likewise, the cavitation process allows obtaining smaller NE due to the implosion of the cavitation bubbles generated in the droplets, breaking them into nanodroplets [21].

#### 3.4.2. Transmission Electron Microscopy

Transmission electron microscopy (TEM) was used to visualize the size and morphology of NE [48,49]. The micrograph obtained by TEM using the negative contrast coating technique is shown in Figure 3b. NECh-LUT showed spherical structure and Dm consistent with the results obtained by DLS. Other authors have also observed the spherical shape and similarity between the Dm determined by DLS and TEM for NE samples. Hong et al. presented similar results from images of NE coated with carboxymethyl chitosan [50]. The authors attributed the spherical appearance of the NE droplets to the lowering of the surface tension offered by the surfactants. Iqbal et al. also used phosphotungstic acid as a negative contrast to visualize NE, verifying Dm between 20 and 200 nm [51]. Likewise, 100 nm droplets of Ch-coated NE were visualized using phosphotungstic acid as negative contrast [52]. Fachel et al. imaged droplets of Ch-coated nanoemulsion of 200 to 300 nm with sharp edges using uranyl acetate negative contrast [41].

#### 3.4.3. Small-Angle X-ray Scattering

**Figure 4:**
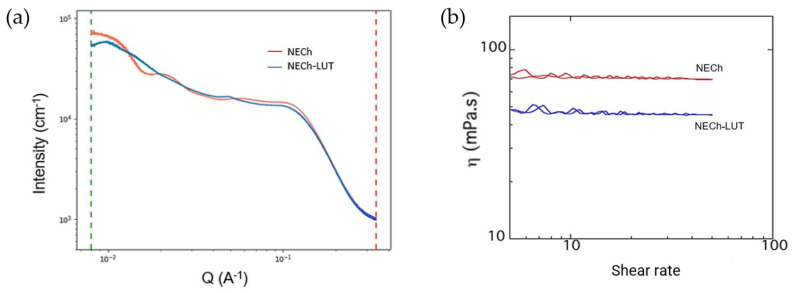
SAXS (**a**) and Rheology (**b**) for NECh-LUT and NECh formulations.

Small angle X-ray scattering (SAXS) measurements are probing the size, size distribution, and shape of nanoparticles [53]. In recent years, the development of synchrotron beamlines, low divergence, low parasitic scattering, and high flux, combined with the development of new detectors, has allowed the characterization of liquid materials such as NE [54]. The use of synchrotron radiation provides orders-of-magnitude increases in brightness over lab sources, reducing acquisition time and sample volume [55]. Compared to TEM, where the field of view or area sampled is greatly decreased to reach nanometer level spatial resolution, SAXS results are integrated over the entire beam foot print (~mm^2^) [53]. SAXS measurements of NECh and NECh-LUT are shown in Figure 4a. The most notable feature of the SAXS patterns of both samples is they display two Guinier regions, suggesting each sample contains two size populations of nanodroplets. This observation is in agreement with DLS measurements, which show bimodal size distributions. The most reasonable fit was obtained modeling two spherical droplets as a dilute system, the first with 27.0 ± 2.6 nm and the second with 1.4 ± 0.26 nm. The first population is probably the smaller of the observed by DLS. The second particle of very small size is likely of Tween 20 micelles [56]. According to Podlogar et al., the NE scattering profile is a result of the ratios of oil, surfactants, and water, where high water contents lead to broad, high intensity peaks [57]. The fact that the NE are not purified and there are three populations of particle sizes makes the SAXS data interpretation challenging.

#### 3.4.4. Rheology

Rheological analyses demonstrate the changes that occur in the viscoelasticity of the samples, measuring the resistance against deformation and directly influencing the formation of NE [58,59]. NECh and NECh-LUT presented a Newtonian behavior, where the viscosity is independent of the applied shear rate. As shown in Figure 4b, the ascending and descending shear slopes are superimposed, indicating that there is no thixotropy in the sample, that is, the shear time does not affect the viscosity. The results obtained are in agreement with the optimal shear rate for application in the bloodstream, with the NECh-LUT showing a slightly lower viscosity than the NECh [58]. This deviation may be related to the slight Dm difference between the formulations. The cavitation process results in a reduction in the viscosity of NE, decreasing Dm and PDI, improving the characteristics of the system [59].

### 3.5. Stability Studies

**Figure 5:**
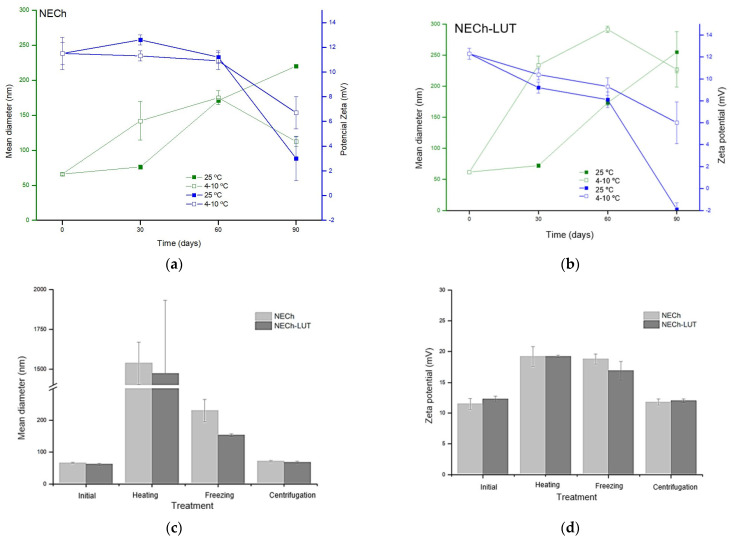
Storage stability at room and refrigeration temperature of NECh (**a**) and NECh-LUT (**b**) regarding mean diameter and Zeta potential. Thermal and kinetic stability of NECh-LUT and NECh regarding mean diameter (**c**) and Zeta potential (**d**).

Stability studies determine the ability of the NE to maintain their physicochemical characteristics during storage time and in conditions that may happen such as increase or decrease in temperature and agitation during transport [49]. NE stability is affected by the components used for its preparation, particle size, and surface charge of the globules [60,61]. The capacity and amount of surfactants added to the NE contributes to the formation of an interfacial film, reducing the surface tension between the aqueous and oil phases, being decisive in the stability of NE [62]. Additionally, smaller and uniform particles are less likely to undergo separation that leads to destabilization [60]. The surface charge, in turn, affects the stability of NE through the electrostatic interactions of repulsion or attraction between the particles, and the electrostatic attraction of NE leads to the flocculation process [35,62]. The storage stability study of NECh and NECh-LUT was carried out by measuring Dm and ZP after 30, 60, and 90 days of storage at room and refrigerated temperature (Figure 5a,b). Likewise, kinetic and thermal stability were evaluated through Dm and ZP evaluation after centrifugation, heating, and freezing treatments (Figure 5c,d).

The translucent and uniform appearance of NECh and NECh-LUT was verified throughout the storage stability period. NECh and NECh-LUT did not show a significant difference in Dm and ZP after 30 days of storage at room temperature, (*p* < 0.05). On the other hand, the refrigerated storage led to the destabilization of NECh and NECh-LUT, whose Dm increased from 66.1 to 141.9 nm and from 61.8 to 233.8 nm (*p* < 0.05), respectively, after 30 days. In both storage conditions, NECh showed constant ZP for 60 days, while NECh-LUT maintained ZP for only 30 days (*p* < 0.05). During storage, NE tends to undergo destabilization processes, which can occur through flocculation, coalescence or Ostwald ripening mechanisms [48]. Flocculation and coalescence occur through the interaction between NE droplets. When the electrostatic repulsion between droplets is low, they tend to stick together, forming aggregates that lead to flocculation. Likewise, when the intermolecular forces between the particles are too strong, the interfacial membrane of the particles rupture occurs, leading to coalescence [49]. The destabilization of NE might also occur by Ostwald ripening [48]. The NE stability provided by Ch has been studied by several authors. Silva et al. obtained similar results for the stability of NE containing curcumin and stabilized by Ch, which maintained the characteristics for 35 days [40]. Ch-coated NE prepared by Mistry et al. to encapsulate curcumin showed stability for 60 days, while Hong et al. produced NE coated by carboxymethylchitosan with 90-day stability [50,63]. Dammak and Sobral studied the effect of different biopolymers on the stability of NE containing hesperidin, noting that Ch provided greater stability to emulsified systems than other materials [64]. In fact, Ch provides NE stabilization through steric hindrance and electrostatic repulsion.

Regarding thermal stability, both NECh and NECh-LUT increased in Dm and ZP after heating and freezing processes (*p* < 0.05). During heating process, the NECh and NECh-LUT Dm increased from 66.1 to 1536.8 nm and from 61.8 to 1472.1 nm, respectively, while PZ differed for higher values after heating (*p* < 0.05). Freezing led to an increase in Dm and ZP of both formulations (*p* < 0.05). Dm increased from 66.1 to 230.5 nm and from 61.8 to 153.6 nm in NECh and NECh-LUT, respectively. ZP increased from +11.5 to +18.8 mV and from +12.3 to +16.9 mV for NECh and NECh-LUT, respectively. On the other hand, both NECh and NECh-LUT were kinetically stable after the tests performed, not undergoing significant changes Dm and ZP (*p* < 0.05). In fact, NE are recognized for their kinetic stability and for thermal instability [61]. The kinetic stability of NE is attributed to the resistance to collisions due to the Brownian motion of the droplets, while heating causes the increase in oil phase in the aqueous phase, leading to instability by the Ostwald ripening process [65,66]. Patel and Parikh noted that NE produced with Tween 20 did not show thermal stability [67]. Likewise, NE stabilized for 3 months with carboxymethyl chitosan produced by Hong et al. also did not resist the heating process [50]. In contrast, the NE containing ezetimibe showed thermal stability at temperatures of 30, 40, and 50 °C [34]. Iqbal et al. evaluated the stability of NE containing letrozole in different proportions of oil and surfactant, verifying the thermal stability of formulations with proportions greater than oil:surfactant 1:5 [51]. However, the amount of surfactants in NE must be optimized, considering the toxicity of these components when used in large amounts [44].

### 3.6. In Vitro Release

**Figure 6:**
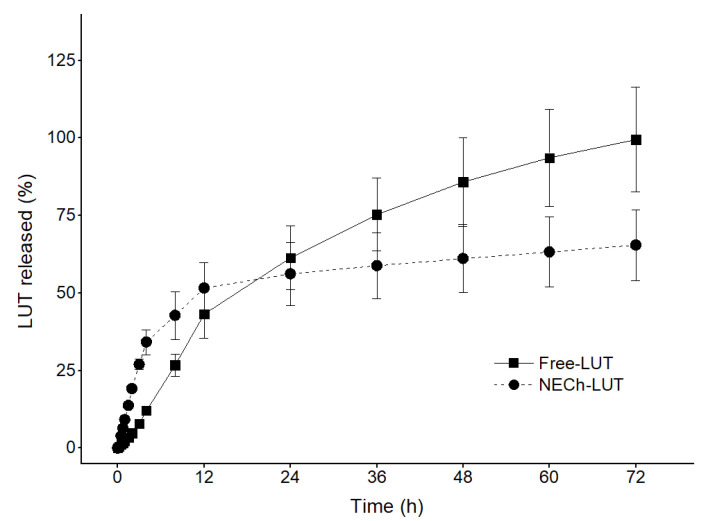
In vitro release profile of LUT from Free-LUT and NECh-LUT during 72 h of assay.

Figure 6 shows in vitro release profile of free LUT in suspension (Free-LUT) and NECh-LUT. A prolonged release pattern was observed for NECh-LUT. In the first 12 h, NECh-LUT presented an initial rapid release, a burst effect, in which about 50% of the drug was released. The burst effect is due to the release of drug that was not completely incorporated into the NECh-LUT or absorbed on the formulation surface. After 12 h, the release reached a steady state phase, leading to the accumulation of 65.47% at the end of 72 h, demonstrating a prolonged and sustained release. Free-LUT also demonstrated a rapid initial release, reaching 43.22% of accumulated LUT after 12 h. However, contrary to NECh-LUT, Free-LUT presented a maintained release pattern, accumulating 75.35 and 99.51% of drug released after 36 and 72 h, respectively.

Natesan et al. obtained similar results of camptothecin release from chitosan-stabilized NE with Dm of 64 nm, characterized by an initial burst followed by a sustained release for a long period, reaching 61.65% of drug released after 24 h [68]. Drug release from NE was attributed to the formed structure and diffusion capacity of the oil core in the surfactant layers. On the other hand, Fachel et al. observed that rosmarinic-acid-containing NE released 78.4% of the drug after 8 h [41]. Singh et al. demonstrated that the nano size of NE droplets decreased drug diffusion time, leading to faster release [47]. The authors also suggest that the NE coating employing polymers such as chitosan provide controlled release. Similarly to our study, Shao et al. employed chitosan coating as a means of controlling the release of Eugenol from NE, which showed an initial burst effect, followed by a slow sustained release, reaching 55% of drug released after 168 h [69]. Barradas et al. state that chitosan-coated NE presents a greater density of intermolecular aggregates, and consequently, higher viscosity [70].

The obtained results are described in Table 7 and reveal the best fit of the data for the Weibull model for Free-LUT (r^2^ = 0.995) and NECh-LUT (r^2^ = 0.986). The Weibull release model is an empirical and flexible model with great potential to be adjusted to a variety of release patterns, being widely used for immediate and sustained drug release. The Weibull model is expressed by Equation (1), where *M*(*t*)/*M_∞_* corresponds to the accumulated fraction of the drug in solution as a function of *t*, a represents the scale parameter, *Ti* is the interval before the start of release, and parameter *b* indicates the shape of the curve obtained. When *b* < 1, the curve is parabolic, while *b* = 1 describes an exponential behavior and *b* > 1 a sigmoid curve. The value of b also indicates the drug release mechanism, which can occur through simple diffusion (*b* < 0.75), diffusion described by more than one release mechanism (0.75 < *b* < 1) or anomalously (*b* > 1).

(1) $$M\left(t\right)/M_{\infty }=1-\exp\left[-\frac{{\left(t-T_{i}\right)}^{b}}{a}\right]$$

The values of *b* obtained for LUT release from Free-LUT and NECh-LUT (1.0 and 1.2, respectively) indicate that the release has occurred through Fickian diffusion from Free-LUT and through other contributing complex mechanisms from NECh-LUT.

## 4. Conclusions

A chitosan-based nanoemulsion was developed for LUT encapsulation. The 2^3^ factorial design employed was efficient in determining the proportions of oil, water, and surfactants to produce NE of adequate mean diameter and polydispersity index. The obtained NECh-LUT system presented mean diameter, polydispersity index, surface charge, and viscosity that corroborate the characteristics of NE with the ability to transpose membranes. TEM analysis showed spherical characteristics and the rheological analysis verified the Newtonian behavior of NECh-LUT, while SAXS technique confirmed its bimodal characteristic. NECh-LUT demonstrated kinetic and storage stability at room temperature for up to 30 days and proved controlled release of LUT through the diffusion process. The results obtained demonstrate the potential of NECh-LUT as a means of controlled released LUT.

## Figures and Tables

**Table 1:** Independent variables of the 2^3^ factorial design for NE optimization.

Codified Independent Variables					Real Independent Variables	
Assay	X1	X2	X3	Oil (mL)	Aqueous Phase (mL)	Surfactants (mL)
1	−1	−1	−1	0.5	3.0	1.0
2	−1	−1	+1	0.5	3.0	2.0
3	−1	+1	−1	0.5	5.0	1.0
4	−1	+1	+1	0.5	5.0	2.0
5	+1	−1	−1	1.0	3.0	1.0
6	+1	−1	+1	1.0	3.0	2.0
7	+1	+1	−1	1.0	5.0	1.0
8	+1	+1	+1	1.0	5.0	2.0

**Table 2:** Results of mean diameter and polydispersity index obtained in each test of the 2^3^ factorial design.

Formulation	Oil (mL)	Aqueous Phase (mL)	Surfactants (mL)	LUT (mg/mL)	Mean Diameter (nm)	Polydispersity Index
1	0.5	3.0	1.0	0.8	810.2 ± 77.8 ^b,c^	0.902 ± 0.07 ^a^
2	0.5	3.0	2.0	0.8	385.1 ± 27.1 ^d^	0.381 ± 0.03 ^b^
3	0.5	5.0	1.0	0.8	146.0 ± 15.7 ^e^	0.805 ± 0.07 ^a^
4	0.5	5.0	2.0	0.8	39.7 ± 0.5 ^f^	0.163 ± 0.01 ^d^
5	1.0	3.0	1.0	0.8	2389.7 ± 186.5 ^a^	0.975 ± 0.04 ^a^
6	1.0	3.0	2.0	0.8	921.9 ± 52.8 ^b^	0.412 ± 0.06 ^b^
7	1.0	5.0	1.0	0.8	612.2 ± 26.7 ^c^	0.508 ± 0.07 ^b^
8	1.0	5.0	2.0	0.8	418.7 ± 8.2 ^d^	0.273 ± 0.02 ^c^

^a, b, c, d, e, f^ Means followed by the same letter in the column do not differ from each other by Tukey’s test (*p* < 0.05).

**Table 3:** ANOVA results for the dependent variables mean diameter and polydispersity index.

Factors	Mean Diameter				
	Sum Squares	DF	Mean Squares	F-Calc	*p*-Value
Oil (X1)	3,288,635	1	3,288,635	129.0678 *	0.000000 *
Aqueous phase (X2)	4,059,613	1	4,059,613	159.3261 *	0.000000 *
Surfactants (X3)	1,803,085	1	1,803,085	70.7650 *	0.000000 *
X1:X2	605,886	1	605,886	23.7790 *	0.000142 *
X1:X3	478,866	1	478,866	18.7939 *	0.000450 *
X2:X3	951,658	1	951,658	37.3494 *	0.000012 *
Residue	433,158	17	25,480		
Total	11,620,901	23			
	**Polydispersity index**				
Oil (X1)	0.012604	1	0.012604	2.3720	0.141932
Aqueous phase (X2)	0.252150	1	0.252150	47.4533 *	0.000003 *
Surfactants (X3)	1.300142	1	1.300142	244.6797 *	0.000000 *
X1:X2	0.057624	1	0.057624	10.8445 *	0.004294 *
X1:X3	0.026268	1	0.026268	4.9435 *	0.040035 *
X2:X3	0.035574	1	0.035574	6.6948 *	0.019171 *
Residue	0.090332	17	0.005314		
Total	1.774694	23			

Values followed by * present statistical significance (*p* < 0.05).

**Table 4:** Models generated by multiple linear regression considering the significant effects for the mean diameter and polydispersity index responses.

Generated Model	R^2^
$\mathrm{Mean}\;\mathrm{diameter}=715.446+0.532x_{1}-0.591x_{2}-0.394x_{3}$	0.756
$\mathrm{Polidispersity}\;\mathrm{index}=0.565-0.377x_{2}-0.856x_{3}$	0.864

**Table 5:** Dm, PDI, and PZ results obtained according to the concentration of Ch added to NE.

Formulation	Chitosan (%)	Mean Diameter (nm)	Polydispersity Index	Zeta Potential (mV)
4	-	39.7 ± 0.5 ^d^	0.163 ± 0.010 ^b^	−3.7 ± 0.67 ^e^
9	0.10	55.3 ± 3.2 ^d^	0.136 ± 0.006 ^c^	+3.2 ± 0.02 ^d^
10	0.25	67.5 ± 3.3 ^c^	0.174 ± 0.005 ^b^	+12.8 ± 0.04 ^c^
11	0.50	261.2 ± 15.9 ^b^	0.294 ± 0.013 ^a^	+16.2 ± 0.03 ^b^
12	1.00	378.5 ± 20.8 ^a^	0.334 ± 0.030 ^a^	+25.3 ± 0.10 ^a^

^a, b, c, d, e^ Means followed by the same letter in the column do not differ from each other by Tukey’s test (*p* < 0.05).

**Table 6:** Mean diameter, polydispersity index, and Zeta potential results obtained for nanoemulsion formulations.

Formulation	Mean Diameter (nm)	Polydispersity Index	Zeta Potential (mV)
NECh-LUT *	67.5 ± 0.7 ^a^	0.174 ± 0.002 ^a^	+12.8 ± 0.64 ^a^
NE-LUT	39.7 ± 0.3 ^b^	0.163 ± 0.003 ^a^	−3.7 ± 0.67 ^b^
NECh	61.8 ± 1.6 ^a^	0.186 ± 0.007 ^a^	+10.6 ± 0.92 ^a^

^a, b,^ Means followed by the same letter in the column do not differ from each other by Tukey’s test (*p* < 0.05). * NECh-LUT = Chitosan-based NE containing LUT; NE-LUT = NE containing LUT; NECh = Chitosan-based NE without LUT.

**Table 7:** Correlation coefficient values (r^2^) obtained for each kinetic model applied.

Model	Free-LUT (r^2^)	NECh-LUT (r^2^)
Zero order	0.927	0.695
First order	0.636	0.877
Second order	0.215	0.368
Higuchi	0.393	0.118
Hixson–Crowell	0.774	0.463
Weibull	0.995	0.986

## Data Availability

Not applicable.
